# Experimental and DFT study of photocatalytic activity of reduced graphene oxide/copper sulfide composite for removal of organic dyes from water

**DOI:** 10.1038/s41598-023-42680-3

**Published:** 2023-09-20

**Authors:** Mohamed S. Sadek, Ghada E. Khedr, Michel F. Abdel Messih, Mohamed Abdel Hay Ismail

**Affiliations:** 1 Chemistry Department, Faculty of Science, Ain-Shams University, Cairo, Egypt; 2 Department of Analysis and Evaluation, Egyptian Petroleum Research Institute (EPRI), Cairo, 11727 Egypt

**Keywords:** Chemistry, Materials science, Nanoscience and technology

## Abstract

In this work, successful nanocomposites composed of different ratios of reduced graphene oxide and copper sulphide (xCuS–rGO) were fabricated to aid in treating water contaminated with organic dyes. XRD, TEM, SEM, XPS, IR, EDX and BET were applied for the characterization of (CuS–rGO). The photocatalytic strength of the prepared nanocomposites was evaluated using artificial sunlight irradiation. The nanocomposites were tested for their ability to degrade both anionic and cationic organic dyes, including amaranth and rhodamine B (RhB). The excellent photocatalytic strength of our composites, relative to pristine CuS and rGO, was interpreted as rGO sheets being very porous. In addition, the charge moved efficiently from rGO to CuS. The combined properties enhanced the efficiency of photodegradation of CuS–rGO composite across the dyes under the illumination of simulated sunlight. The electron transportation from rGO sheets to the CuS conduction band enhances the charge separation and transportation. The role of superoxide radicals in photocatalytic degradation was unveiled and the interactions between the studied dyes and our catalysts were investigated by density functional theory study and scavenging investigation. This work gives new ideas about the preparation and properties of (CuS–rGO) composites and their broad application in solving environmental problems.

## Introduction

Graphene oxide (GO) is a 2D carbon material with various applications. Recently, It has drawn inconceivable research concern due to its several uses as a standard precursor for giant preparation of reduced graphene oxide (rGO) via chemical or physical treatment for its functional groups^1–4^. rGO has also proven its prospects within the electronics domain and optoelectronics area, thanks to its simple synthesis, cheap fabrication, good conductivity, elasticity, and compatibility with different materials^5–10^. From various physical reduction processes, photocatalytic method is an excellent clean technique for GO reduction by semiconductor with tunable band gap^11–13^. It controls the GO reduction in a good manner, that helps in adjusting the band gap^14,15^. In recent years, researchers have been interested in discovering ways to benefit from the wide surface area of rGO nanosheets to load various nanomaterials, creating new composites with unique properties for several up to date applications^16–22^. In contrast, while the reduction operation, nanomaterial is established on the GO, yielding rGO-based composites. Till now, little nanomaterials such as ZnO^11,12^, TiO_2_^23,24^, BiVO_4_^13^_,_ zinc ferrite^25^ have been utilized to reduce GO via irradiation with ultra violet (UV) or visible light. The efficiency of oxide semiconductors is reduced due to oxygen vacancies and absorption–desorption that occur at the surface^26^. Due to the distinctive optical and electrical characteristics of different metal sulfide materials, they are now considered as fundamental building blocks in optoelectronic applications^27,28^. Copper sulfide is considered as one among the main p-type semiconductors owing to its abundance, little poisoning and versatility. Additionally, it has superior electronic, optical and other significant features^29^. Owing to its adequate photosensitivity, superior physical and chemical stability, CuS is a leading semiconducting nanomaterial which has direct band gap with several prospective applications. These applications involve photothermal conversion, lithium ion batteries, gas sensing, nanometer-scale switches, catalysis and solar cell^30^. Lately, CuS has played a potentially significant role in the degradation of dyes, which are considered to be the most common environmental contaminants, due to its photocatalytic performance^31^. So, it is a good idea to fabricate a composite from CuS and rGO. To our best knowledge, few researchers have prepared CuS–rGO composite to overcome the drawbacks of CuS by adding small ratios of rGO. For example, Y. Wang et al. reported that CuS with small ratios of Gr had better photocatalytic activity towards methylene blue dye than pure CuS^32^. SivaKarthik et al. showed that CuS–rGO has higher photoefficiency in dye sensitized solar cell (DSSC) than pure CuS^33^. In this work we introduced new idea as we enhanced the catalytic activity of rGO by using various ratios from CuS. The catalytic efficiencies for the synthesized CuS–rGO catalysts towards degrading amaranth and RhB dyes were investigated. Amaranth is an example of an anionic dye used in the manufacturing of paper, leather and fiber. It has absorption peak at around 520 nm and is a known carcinogen. Amaranth has been linked to allergic reactions in some individuals, especially those sensitive to azo dyes. Allergic reactions can manifest as skin rashes, hives, itching, and in severe cases, anaphylactic reactions. Some studies, particularly in the context of children, have raised concerns about the potential for certain food colorants, including Amaranth, to exacerbate hyperactivity and behavioral issues. RhB is an example of a cationic dye which is hydrophilic and widely applied in the industrialization of pharmaceuticals, printing paper, food products and textiles^34,35^. It is also well-known as a sensor for water fluorescence. It causes damage to the respiratory system, eyes and skin. It may have adverse health effects. RhB has been classified as a potential carcinogen by some regulatory agencies due to its chemical structure and potential genotoxicity. Long-term exposure to high concentrations of RhB has been associated with an increased risk of cancer in experimental animals. Ingesting large amounts of RhB can lead to various adverse health effects. It may bring gastrointestinal disturbances involving vomiting, nausea, diarrhea and abdominal pain. It is known to have sensitizing properties, which means it can cause allergic reactions when it comes into contact with the skin^36^. Skin irritation and contact dermatitis are possible outcomes. Direct contact with RhB can cause irritation to the eyes, leading to redness, tearing, and discomfort.

There is some evidence suggesting that exposure to high levels of RhB might have adverse effects on reproduction and development. RhB is not only potentially harmful to humans but also can be harmful to the environment. It can be toxic to aquatic organisms and persist in water bodies, affecting aquatic ecosystems^37^. Due to their potential health risks, the use of amaranth and rhodamine B in food and cosmetics has been restricted or banned in many countries. Regulatory agencies like the European Chemicals Agency (ECHA) and the United States Food and Drug Administration (FDA) have ruled restrictions on their use in certain products. Hence, the removal of dye wastewater is a serious problem that needs to be considered all over the world. In addition to the experimental work, density functional theory (DFT) calculations were carried out for more clarification of the mechanism of dye degradation. DFT plays a substantial role in recognizing material properties^38–40^, reaction mechanisms^41–46^ and optics^47,48^.

## Experiment

### Materials

Graphite, sodium Nitrate [NaNO_3_], hydrazine hydrate, copper nitrate, rhodamine B [RhB], amaranth and potassium permanganate [KMnO_4_] were bought from Sigma-Aldrich. Sulphuric Acid [H_2_SO_4_], sodium sulphide, hydrogen Peroxide [H_2_O_2_], hydrochloric Acid [HCl] were bought from Merck. All the chemicals were utilized, as extradited.

### Preparation of rGO–CuS nanocomposite

Graphene oxide was prepared according to the modified Hummers’ recipe^49,50^. GR was synthesized by reducing GO using hydrazine hydrate applied through the modified Hummers recipe as described: GO (200 mg) was loaded into a 500-ml round-bottom flask. Then, we added 200 ml water, producing an inhomogeneous yellow–brown product. The solution was sonicated until it became pure with no apparent particulate matter. Afterward, we added 1 ml of hydrazine hydrate followed by heating the mixture at 100°C using a water-cooled condenser for one day. During this time, the rGO precipitated out gradually in the form of a black matter. We then filtered the precipitate and washed it profusely with water (5 × 100 ml) and methanol (5 × 100 ml) before desiccating it at 60°C to get graphene powder. The preparation of copper sulfide followed a typical method and was performed as follows: 1g Cu(NO_3_)_2_ 2.5H_2_O was added to 125 cm^3^ of HOCH_2_CH_2_OH and stirred by magnetic stirrer. Then, 1.1 g of Na_2_S· 9H_2_O was dissolved in 25 cm^3^ of HOCH_2_CH_2_OH in a separate beaker. We selected these amounts to keep the molar ratio between Copper and Sulphur to be 1. We put both solutions into a flask then stirring for 2h at 80℃. A precipitate was noticed within the solutions after completing the reaction. We centrifuged the solutions for 25 min at 1100 rpm and washed them several times with distilled water and absolute ethanol and finally the product was dried at room temperature. Series of rGO–CuS nanocomposites were prepared by blending different concentrations from CuS and rGO. 0.1 g CuS and 0.9 g rGO were put in 500 ml of H_2_O and sonicated for one hour The mixture was then stirred for 90 min and filtered. Finally, the filtrate was dried at 40℃ to prepare 10% CuS–rGO. Different ratios of CuS–rGO, ranging from 5 to 20%, were prepared using the same procedure.

### Computational methods

All calculations were performed using Gaussian 16 software with the hybrid functional B3LYP method and 6–311 + g (d,p) basis sets for C, O and H atoms. The Stuttgart-Dresden SDD basis set was used for Cu and S atoms. Data was visualized using GaussView 6 and ChemCraft software^51^. The graphene sheet was constructed using the Nanotube Modeler package from 180 carbon atoms, with the ends capped with hydrogen atom. The CuS cluster was built from twenty atoms (CuS)_10_ to approach the experimental ratio. The adsorption energy (E_ads_) was calculated using Eq. (1).

1 $$E_{ads} = E_{{\left( {dye + surf} \right)}} - E_{dye} - E_{surf}$$

where *E*_(dye+surf)_ is the energy of the surface and the adsorbed dye, *E*_(dye)_ is the energy of the optimized dye and *E*_(surf)_ is the energy of the surface.

## Characterization

### Material characterization

X-ray powder diffraction (XRD) patterns were performed at 40 kV and 40 mA on a powder X-ray diffractometer within 2θ = 10°–80° using Cu Kα radiation (Model X'Pert pro). The structure morphologies were determined using a high-resolution transmission electron microscope (HRTEM, JEOL 2100, Japan). FTIR transmittance was collected on spectrophotometer (a Nicolet Is 10 FTIR Thermo Fisher Scientific spectrometer), US. XPS was performed using an X-ray photoelectron spectrometer (XPS ESCALAB 250XI, Thermo Scientific). For further characterization, BET, PL, DRS and SEM with EDX were applied.

**Figure 1:**
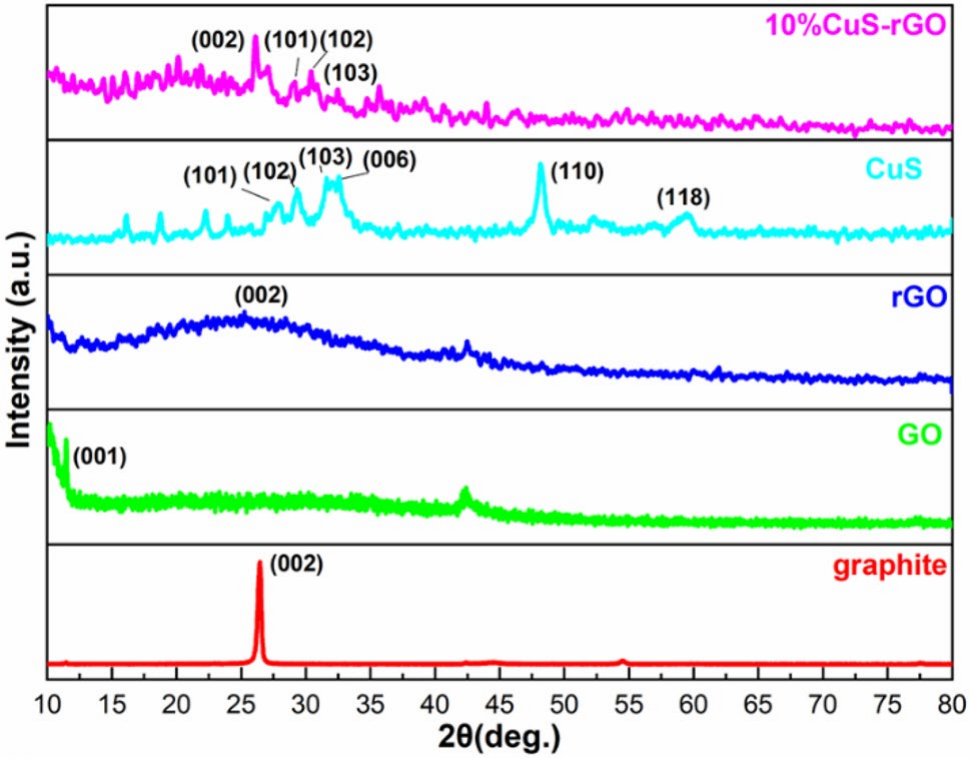
XRD Pattern of graphite, graphene Oxide (GO), reduced graphene oxide (rGO), copper sulphide and 10% CuS–rGO composite.

The XRD pattern of each material is shown for comparison in Fig. 1, with a diffraction peak visible at 26.3° that represents the graphene plane. The (002) peak of graphene is not strong indicates that graphene sheets are not stacked, and the growth of CuS on graphene sheets reduces the stacking of GR sheets. The characteristic peaks for graphene oxide disappeared from the XRD data, indicating the excellent reduction of GO to rGO. The diffraction peaks at 27.6 (101), 29.6 (102), 31.7 (103), 32.8 (006), 47.9 (110) and 59.3 (118) indicating the consistence of pure CuS. In addition, the peak intensity decreases with the inclusion of CuS. When comparing the peaks of CuS in the composite structure to its corresponding bare CuS, it could be observed that the intensity of CuS peaks is reduced due to the presence of graphene oxide. Moreover, the peak intensity of rGO is overly strong, which leads to the comparatively weaker intensity of CuS^52^. This observation highlights the influence of graphene oxide on the diffraction pattern of the composite.

**Figure 2:**
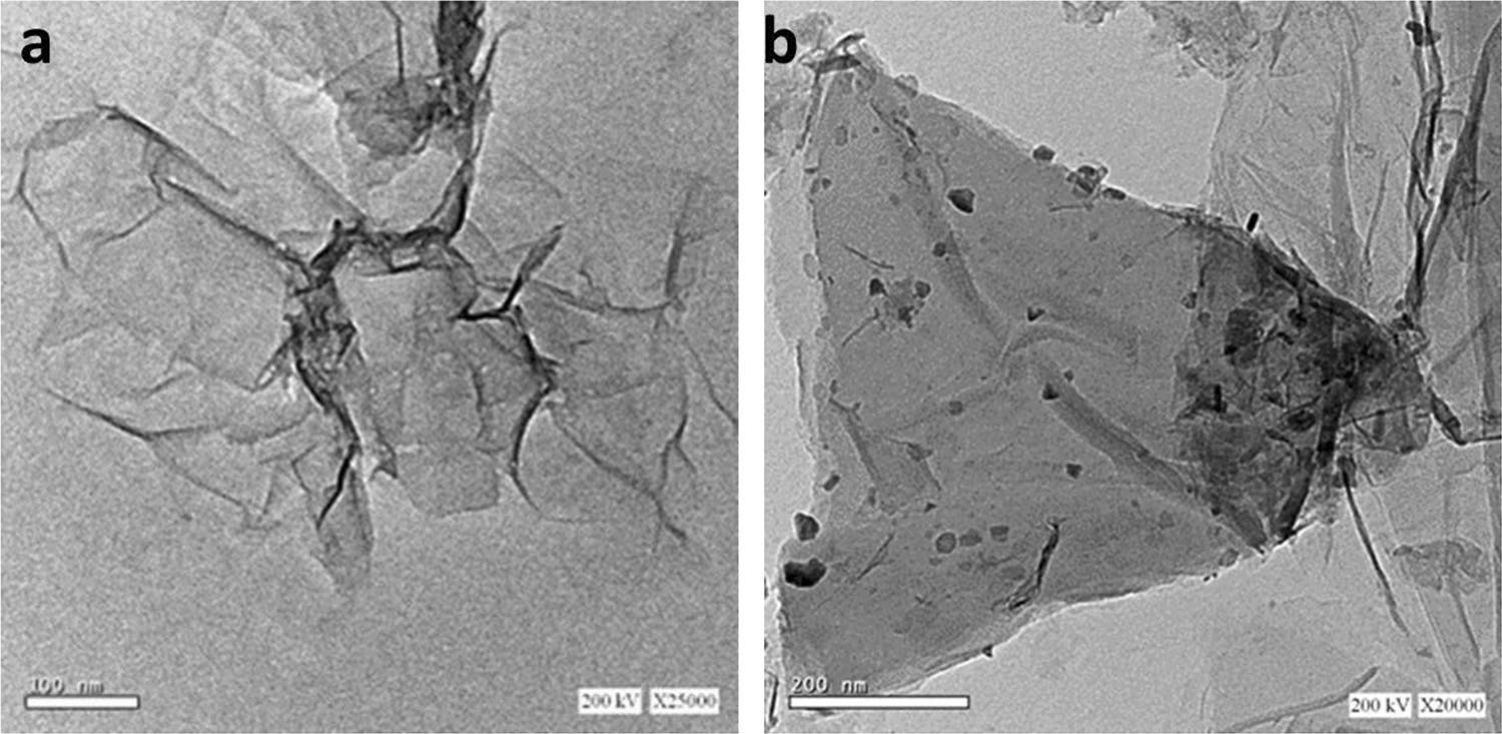
TEM Images of (**a**) rGO Sheets (**b**) 10%rGO/CuS composite.

**Figure 3:**
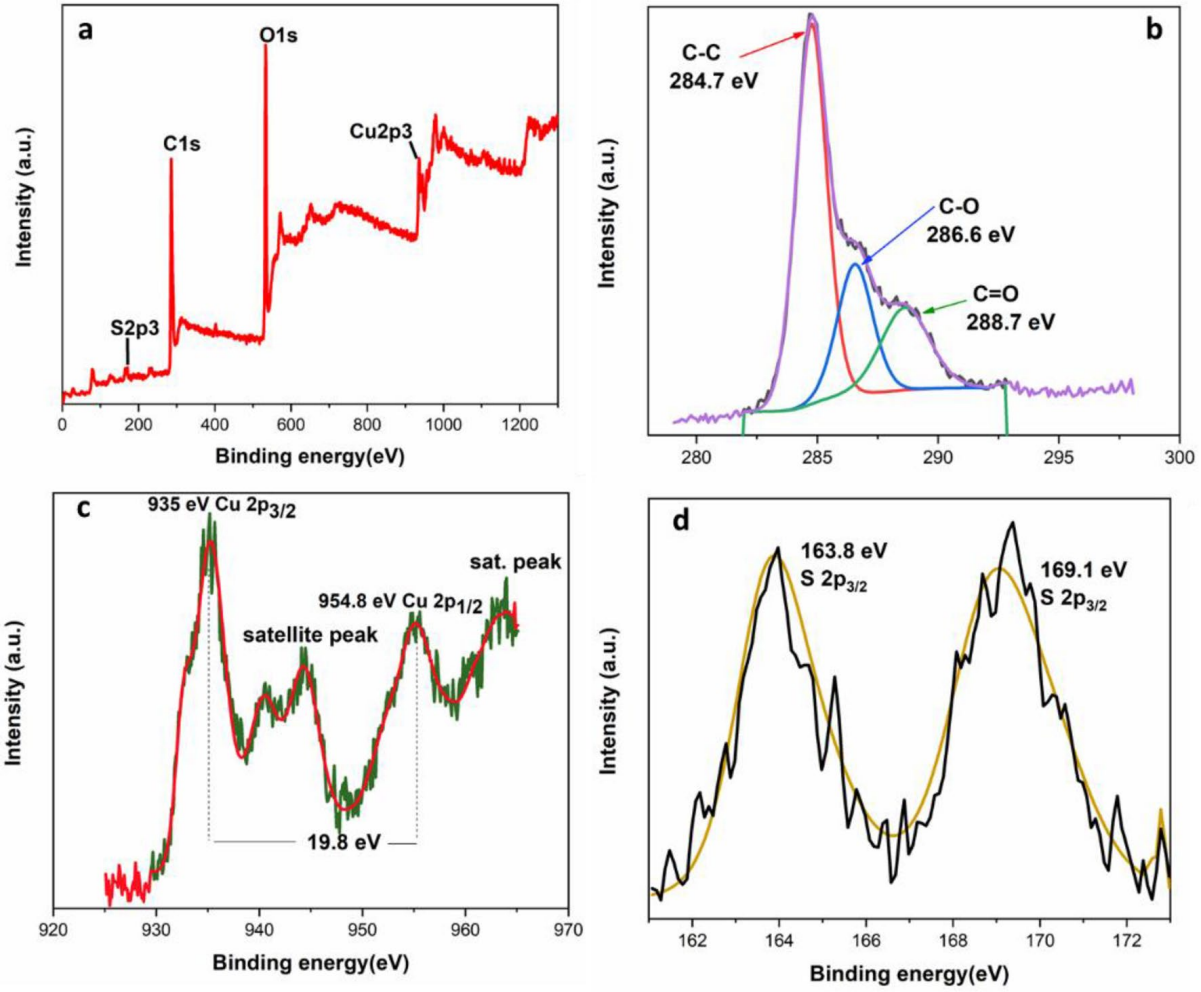
XPS spectra of CuS–rGO composite: (**a**) survey spectra, (**b**) C 1s region XPS spectrum, (**c**) Cu 2p region XPS spectra, (**d**) S 2p area XPS spectra.

**Figure 4:**
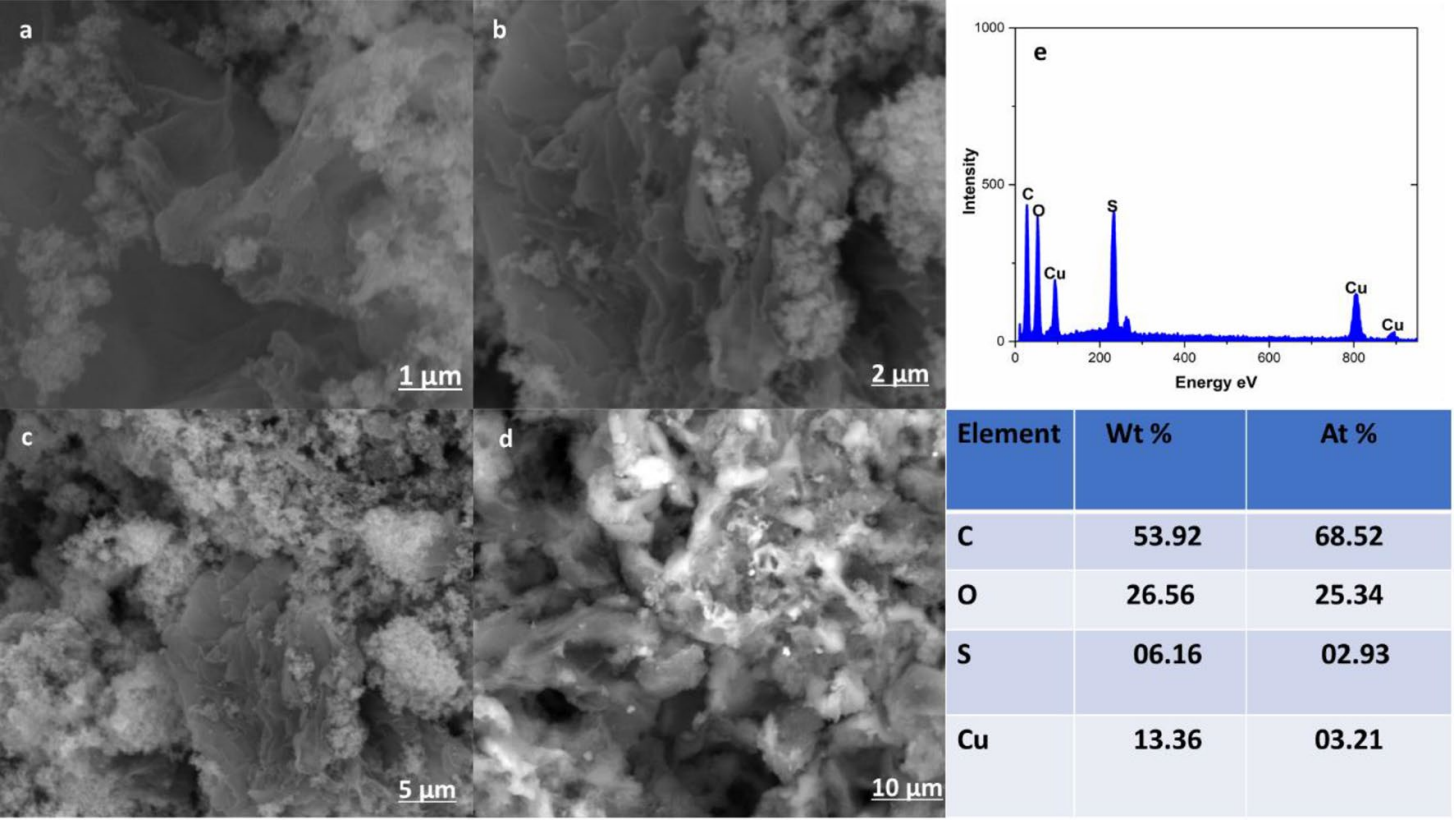
FE-SEM images of CuS–rGO composite at different magnifications (**a**–**d**), (**e**) EDX and weight and atomic percent of CuS–rGO.

Figure 2a, b show the HR-TEM images of CuS–rGO nanocomposites. The TEM results prove the formation of CuS nanoparticles on the rGO sheets. The TEM images assure the combining of CuS nanoparticles with rGO sheets. X-ray photoelectron spectroscopy (XPS) was implemented in the region of 0–1300 eV to investigate the elemental composition and oxidation states of CuS–rGO nanocomposite. As shown in Fig. 3a the wide scan survey of CuS–rGO has peaks at binding energies of 934, 533, 285 and 168.4 eV which are assigned to Cu2p, O1s, C1s and S2p respectively. In Fig. 3b, in the deconvoluted C1s peak, three gaussian peaks are observed at 284.7, 286.6 and 288.7 eV that are related to C–C, C–O and C=O in rGO respectively. Figure 3c exhibits two main peaks at 954.8 and 935.0 eV which correspond to Cu 2p_1/2_ and Cu 2p_3/2_ respectively. The satellite peaks indicate presence of Cu^2+^. The main peaks are separated by spin energy of 19.8 eV. In S2p spectra metal sulfide has a characteristic peak at 162 eV, this value is slightly shifted and broadened as shown in Fig. 3d. This refers to the formation of metal sulphide composite. Also, the binding energy at 169.1 eV is assigned to sulphate. This may be due to the oxidation of sulfur at the surface. XPS data confirmed the presence of CuS and rGO. To investigate the morphology of CuS–rGO composite, FESEM was utilized as shown in Fig. 4a–d. nanosheet-like structure was observed for rGO with uniform nanoparticles of CuS. The energy dispersive X-ray (EDX) data from the SEM images indicates the higher content of carbon in the composite in Fig. 4e.

The optical band gap was estimated according to Eq. (2). In order to estimate the band gap energy, we plotted (*αhυ*)^2^ versus *αυ* as *α* is the absorption coefficient and *hυ* is the energy of photons, A is constant and *E*_*g*_ is the.

band gap.

2 $$\left( {\alpha h\upsilon } \right)^{n} = A\left( {h\upsilon - E_{g} } \right)$$

**Figure 5:**
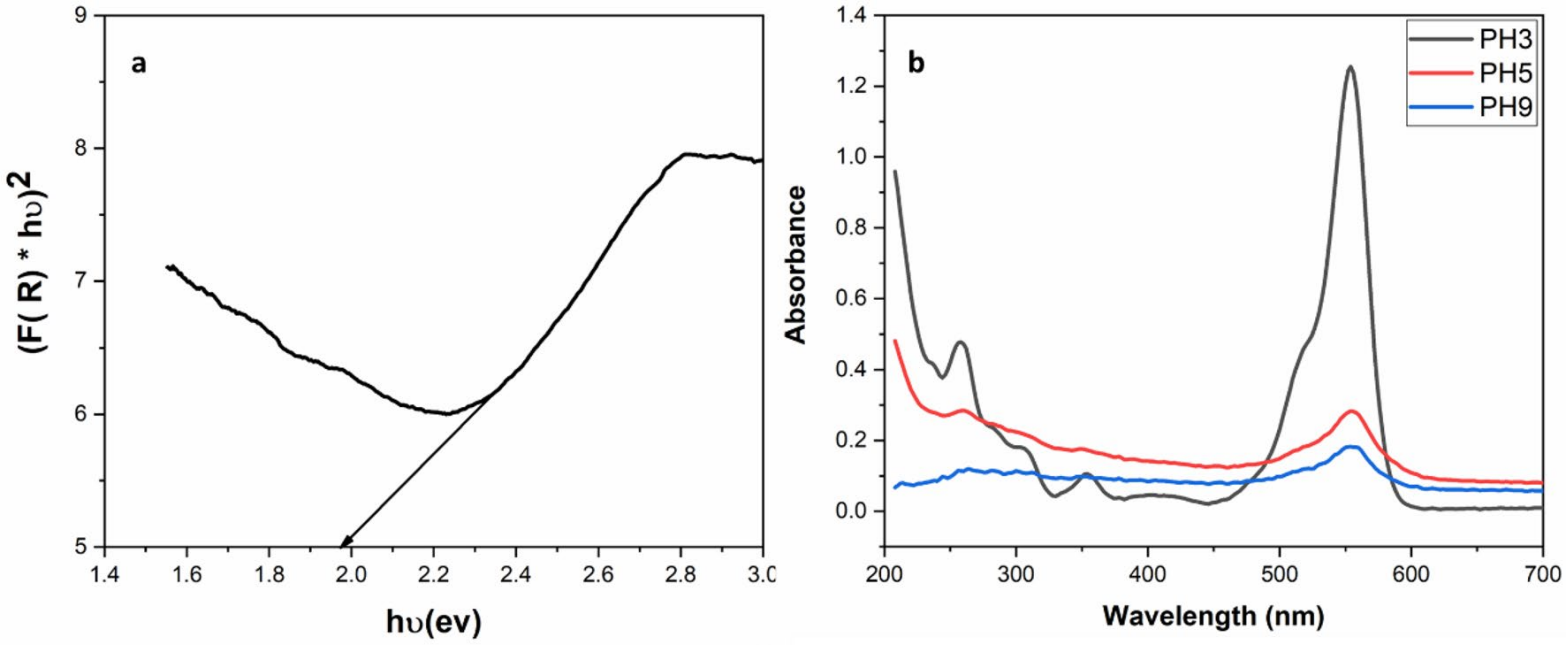
(**a**) Diffuse reflectance spectra (DRS) of CuS. (**b**) UV–Vis absorption spectra for 10%rGO/CuS at different pH values after I h light.

According to Eq. (1) that is focused on direct transitions, the band gap of pure CuS is 1.98 eV as shown in Fig. 5a. Figure 5b shows the UV–Vis absorption spectra for 10%rGO/CuS with RhB dye under different pH conditions 3, 5 and 9 see SI figure S1. As seen the activity of the catalyst affected by solution pH. The photocatalytic degradation increased with an increase in pH value. This is due to the fact that a higher pH enhances the consistence of negative charge on the catalyst, which in turn attracts the positive charge on the cationic dye RhB, resulting in an increase in degradation efficiency for pH 5 and pH 9. On the other hand, at low pH values such as pH 3, there is less degradation because of repulsion between the cationic dye and the positive charge that the catalyst bears^53^.

**Figure 6:**
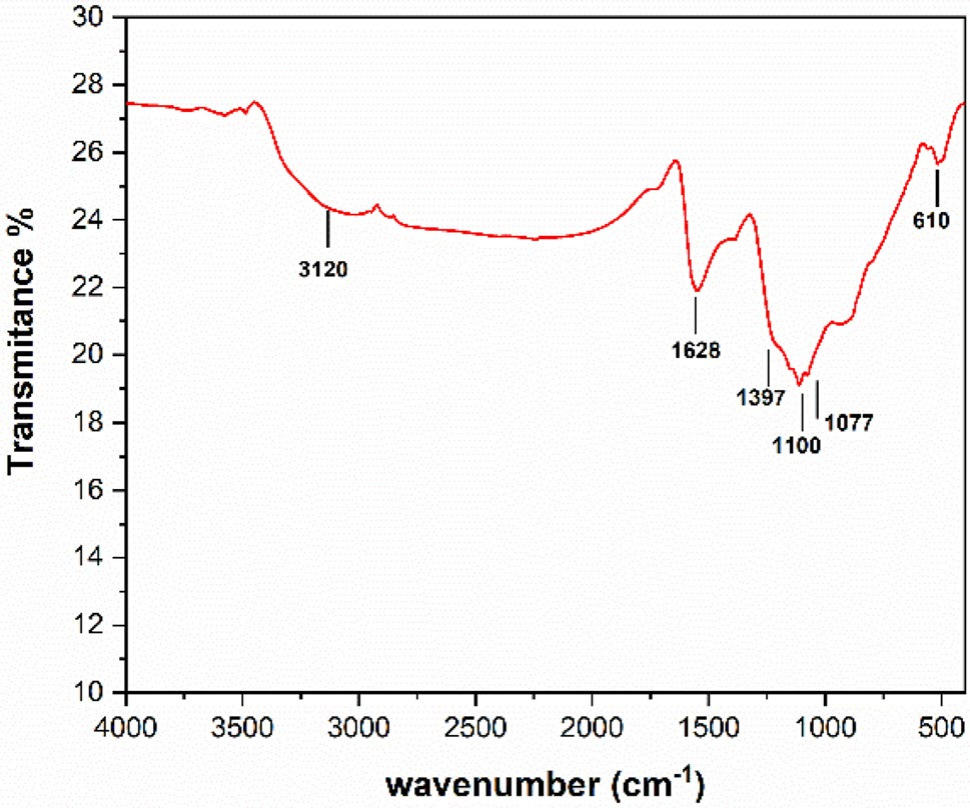
FTIR spectra of 10% CuS–rGO composite.

The FTIR spectrum of CuS–rGO nanocomposites in the 400 cm^−1^ to 4000 cm^−1^ wavenumber range in Fig. 6, shows a low intensity of the absorption peaks for oxygen containing functional group, indicating that GO is partially reduced. The peak observed at around 3120 cm^−1^ is owing to the stretching nodes of the hydroxyl group, whereas the peak observed at 1628 cm^−1^ is due to the O–H bending vibrations mode from absorbed water, C–O–C and the skeletal ring. Additionally, the vibration mode observed at 1399 cm^−1^ corresponds to C–O epoxy and that of C–O alkoxy stretching at 1077 cm^−1^. The strong peak observed at 1100 cm^−1^ refers to C–S stretching, while the peak at 618 cm^−1^ represents the characteristic peak of CuS^32^.

**Figure 7:**
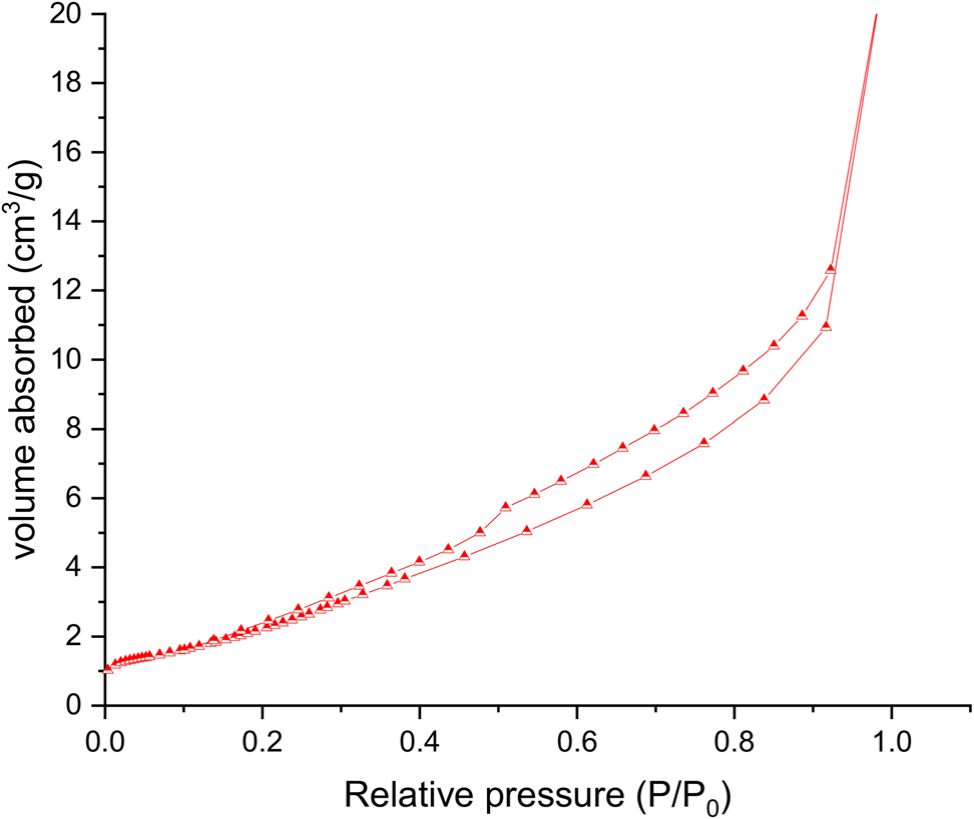
N_2_ adsorption/desorption isotherm of 10% CuS–rGO composite.

Brunauer–Emmett–Teller (BET) technique and N_2_ adsorption–desorption isotherms are employed to determine specific surface area of 10% CuS–rGO as shown in Fig. 7. The specific surface area is 86.9 m^2^ gm^−1^.

**Figure 8:**
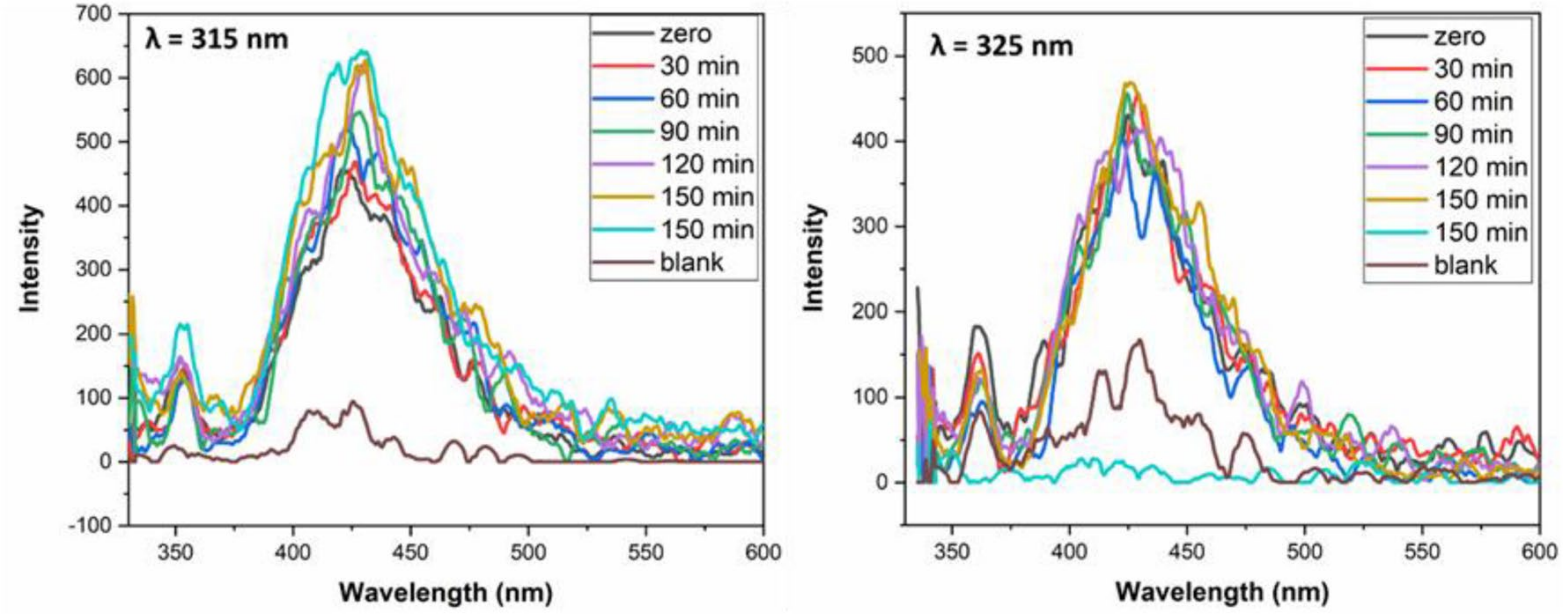
Photoluminescence spectra of 10% CuS–rGO composite in solution of benzoquinone under UV light (λ = 315 nm) at left and (λ = 325 nm) at right side.

**Figure 9:**
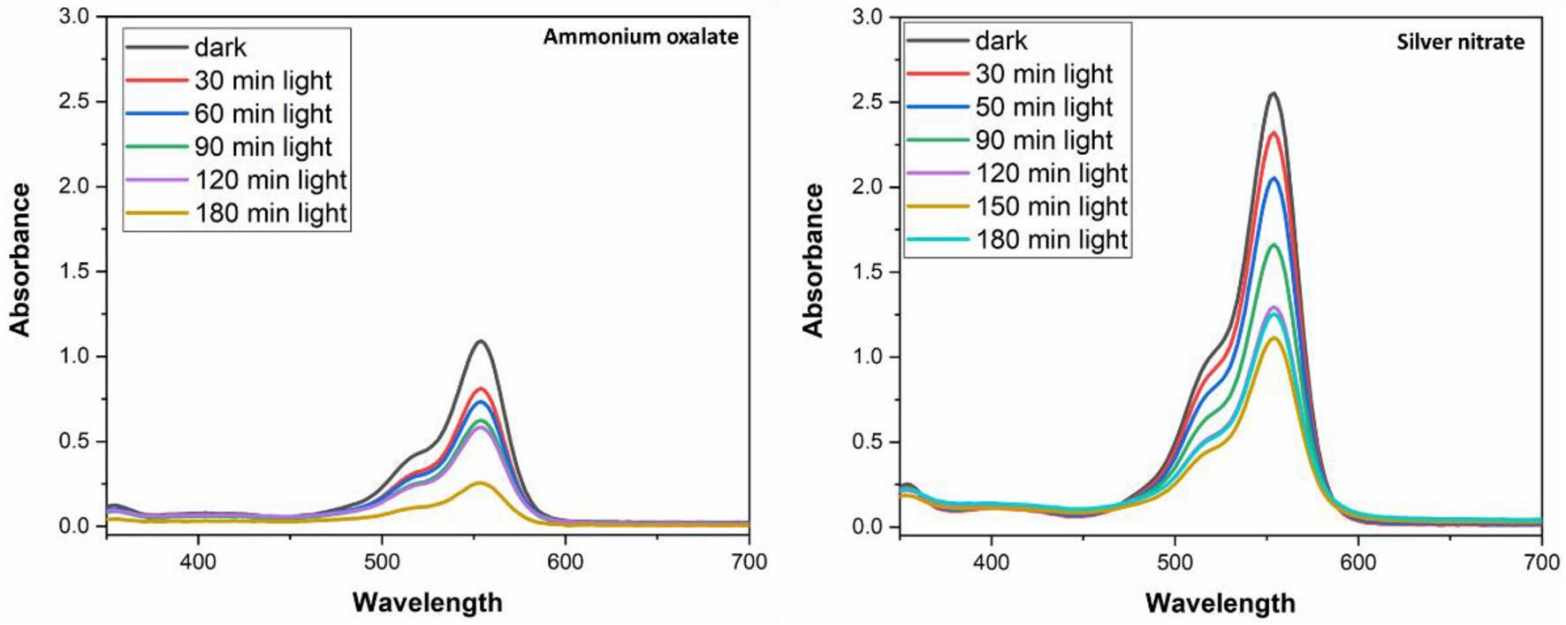
Photocatalytic degradation of rhodamine in the presence of 10% CuS–rGO and scavengers at different times of irradiation.

Photoluminescence (PL) spectroscopy was carried out in aqueous solution containing catalyst with benzoquinone to understand which active species are responsible for dye degradation. The peak intensity refers that O_2_^•−^ radicals were formed in the solution. As cleared in Fig. 8 the PL spectra of 10% CuS–rGO in solution of benzoquinone under excitation by light at λ = 315 nm and at λ = 325 nm. For more clarification to the active species causing the photocatalytic activity, tests were done by adding different scavengers to the catalyst in the aqueous solution. As shown in Fig. 9 ammonium oxalate and silver nitrate were used as hole and electron scavenger respectively. The high intensity peak in case of silver nitrate indicates that the photogenerated electron is the responsible for the degradation process.

### Photocatalytic degradation of rhodamine B in presence of rGO–CuS

**Figure 10:**
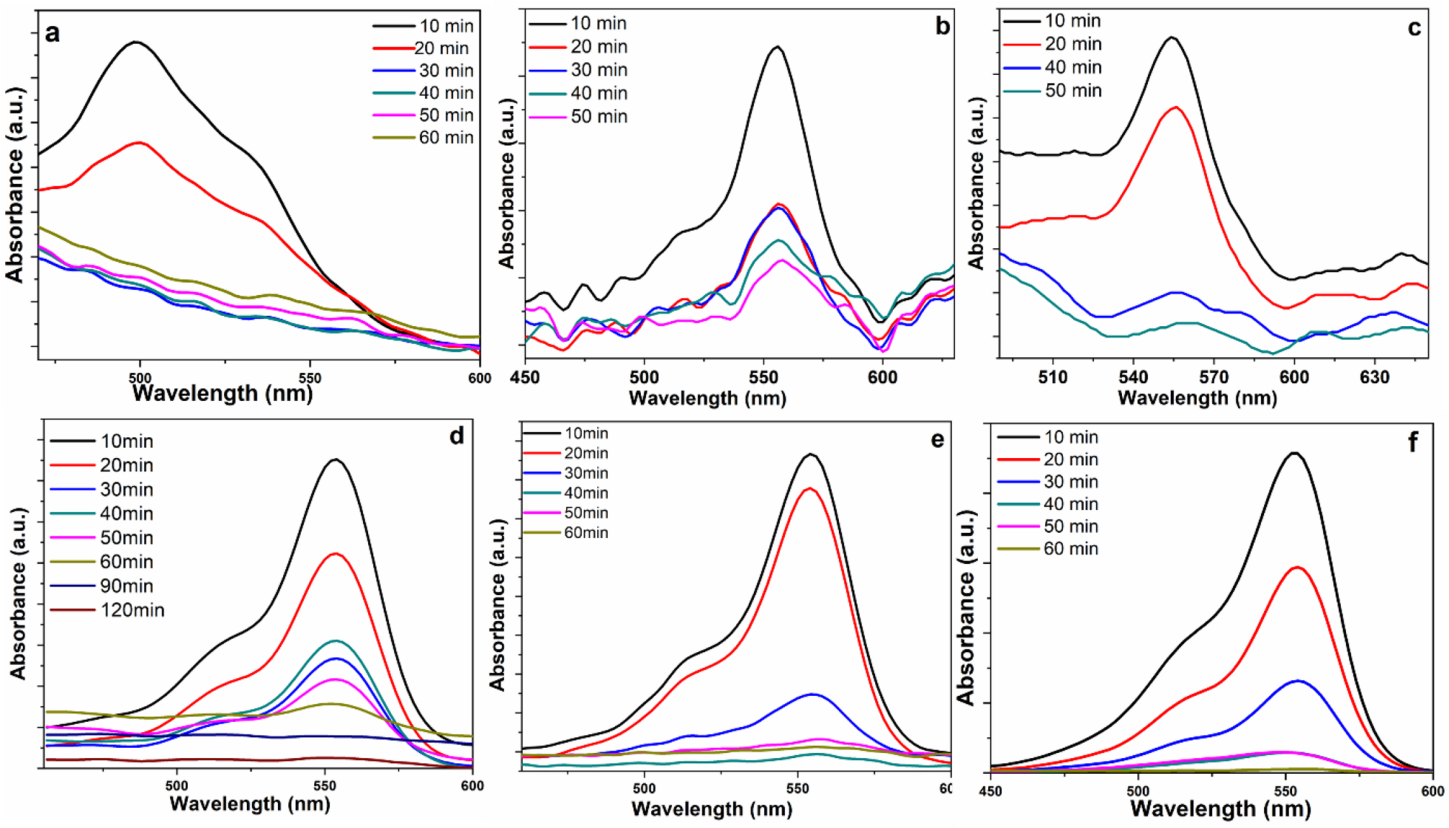
Photodegradation of RhB dye in visible light irradiation using (**a**) rGO, (**b**) CuS, (**c**) rGO with 5% loading of CuS, (**d**) rGO with 7% loading of CuS, (**e**) rGO with 10% loading of CuS, (**f**) rGO with 12% loading of CuS.

Photocatalytic removal of RhB is investigated utilizing local photo‐reactor setup in the range of both UV and visible light. the catalyst was added to the dye and put into dark till adsorption‐desorption equilibrium was reached after that was allowed to receive light. We started with 2*10^–5^ M from the dye. It was noticed that after one hour in dark around 2% to 23% was adsorbed. It was noticed from these results that composite with ratio of 10% CuS has higher affinity for dye adsorption than other ratios. The Fig. 10A displays the optical absorption spectra of RhB solution gathered at definite period of times within the degradation process beneath visible light using graphene as a catalyst while Fig. 10B exhibits the absorption spectra of solution containing RhB compiled at fixed period of times during the degradation procedure within visible light using CuS as a catalyst. The characteristic absorption peak of RhB at 554 nm reduced largely over time. The photocatalytic efficiency of the samples with different concentrations of CuS with graphene (5%, 7%, 10% and 12%) are shown in Fig. 10C–F. The efficiency of the composites can be arranged ascendingly as following rGO < 5% CuS < 7% CuS < 12% CuS < 10% CuS, the curves stated that rGO had the least photocatalytic performance while 10% CuS–rGO had the highest performance among all the studied ratios. The efficiency enhances by raising the CuS concentration from 5% CuS to 10% CuS while more increase in CuS loading to 12% resulted in decreasing in the efficiency. The optimum ratio of CuS combined to rGO is evaluated to be 10% CuS as it achieves efficient dye degradation after one hour at visible light. All the different samples were tested for their photocatalytic performance under UV light at the same experimental conditions. It was obvious that the dye degrades over time ended with color disappearance. It is noticed that 60 min is enough time for efficient dye degradation by our catalyst. Too much amount of CuS limits the photocatalytic efficiency as coverage of rGO with CuS prevented light from reaching the interface of CuS–rGO, in addition, excess CuS acts as the recombination center for the photo‐generated electron‐hole pairs resulted in limitation of photocatalytic efficiency. The present study provides efficient dye removal even under lamps with less wattage (vilber UV lamp made from mercury 365 nm, 12 W) that are cheap and available.

To investigate the removal of RhB dye, we tolerated a reduction in the intensity of the 554 nm absorption peak and estimated it. They show the deviation of the absorption spectrum of RhB at various time periods under visible light. The data exhibits the influence of changing the CuS ratio in the nanocomposites. The peak intensity at 554 nm degenerates with an increase in the time of irradiation, which refers to the removal of RhB. A prompt decline was observed in the intensity of the absorption peak after too much exposure to light, and it totally lapsed after one hour. These figures state that the CuS–rGO nanocomposite containing a CuS ratio of 10% had the greatest photocatalytic efficiency.

### Photocatalytic degradation of amaranth in presence of CuS–rGO

Also, the Photocatalytic efficiency of the prepared composites was evaluated by estimating the degradation of amaranth.

**Figure 11:**
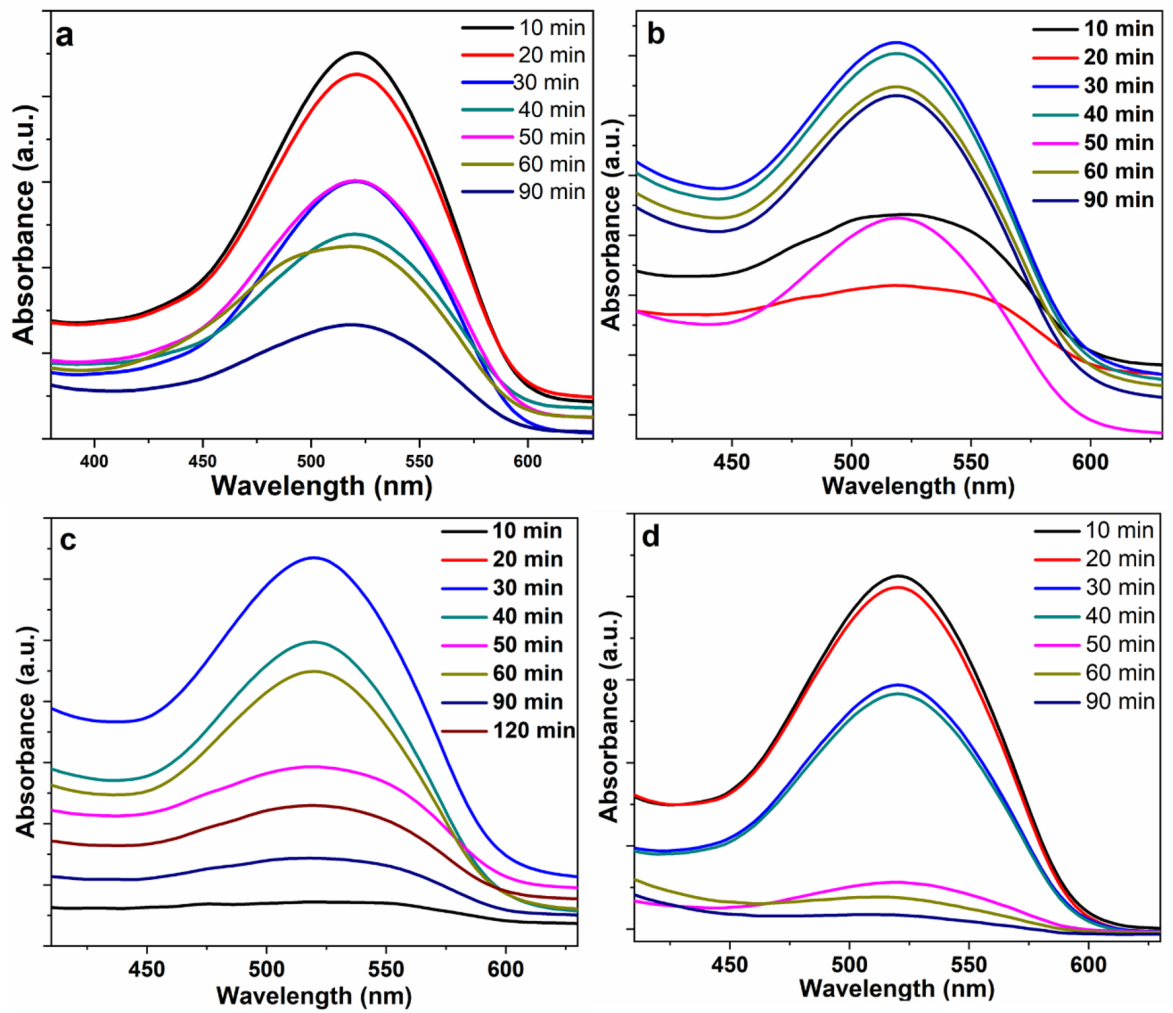
Photodegradation of amaranth dye in visible light irradiation using (**a**) rGO with 10% loading of CuS, (**b**) rGO with 12% loading of CuS, (**c**) rGO with 15% loading of CuS, (**d**) rGO with 20% loading of CuS.

The catalytic efficiencies for the synthesized CuS–rGO catalysts towards degradation of amaranth were studied. The visible light photocatalytic activity of the CuS–rGO composites was estimated by observing the average decolorization of amaranth. Figure 11 shows the photo-degradation of amaranth dye using CuS–rGO nanocomposites, in which the efficiency of adsorption in the dark was found to be 9%, 25%, 29% and 40% for 10% CuS–rGO, 12% CuS–rGO, 15% CuS–rGO, and 20% CuS–rGO nanocomposites, respectively after one hour. After exposure to irradiation, the performance of degrading amaranth dye over CuS–rGO gradually enhanced till 90 min.

Figure 11a–d show the photocatalytic efficiency under light and the adsorption influences in the dark, acquired with different ratios from CuS–rGO. Upon increasing the CuS loading on rGO NSs, the adsorption affinity of rGO NSs was noticed to improve slightly, achieving better performance corresponding to rGO, 10% CuS–rGO, 12% CuS–rGO, 15% CuS–rGO and 20% CuS–rGO respectively, after 90 min the case of amaranth dye. As proven previously in the FTIR discussion, involving polar groups on the surface gives significant exchange capacity to adsorbents and hence improves adsorption of dye. It was stated in literature that rGO acted as electron sensitizer and as donator in the composites. The photo-degradation results indicated that the enhancement in dye degradation of CuS-rGO nanocomposites may be attributed to the improvement of electrons transfer in CuS. when electrons are excited, they yield O_2_^−^ and OH^·^ radicals from oxygen and water molecules, respectively. These reactive species may attack dye molecules which are adsorbed on the surface, thereby improving the rate of dye degradation with increasing time of irradiation for each composite. The percentage of dye degradation elevated as CuS ratio was raised and reached a maximum for amaranth dye when using CuS–rGO nanocomposite (1 gl^−1^ composite dose) after 90 min exposure to light. Table 1 presents a study that compares the efficiency of different catalysts in removing RhB dye through photocatalytic degradation. The results show that the CuS-rGO composite outperforms the other catalysts in terms of photocatalytic activity, which suggests that it could be a highly effective material for removing both RhB and amaranth from aqueous solutions.

**Table 1:** Photocatalytic degradation efficiency of different rGO based photocatalysts.

Photocatalyst	Pollutants	Light source	Initial concentration	Catalyst dose	Irradiation time (min)	Degradation (%)	References
10%CuS–rGO	RhB	UV lamp, 365 nm, 12 W	2.0 × 10^−5^ M	1 gL^−1^	60	98	This work
AgI/rGO (0.4 wt%)	RhB	–	10 ppm	100 mg	70	96	^54^
BiVO4–graphene	RhB	Visible light	20 ppm	50 mg	300	99	^55^
Graphene–CdS	RhB	Visible light	1.0 × 10^−5^ M	20 mg	80	95	^56^
PANI/rGO (rGO-5% wt.)	RhB	Visible light	1.7 × 10^−5^ M		30	99.35	^18^
Ag/AgBr/rGO	RhB	Visible light	1.0 × 10 − 5 M	25 mg	30	87	^57^
20%CuS–rGO	amaranth	UV lamp, 365 nm, 12 W	2.0 × 10^−5^ M	1 gL^−1^	90	95	This work

**Figure 12:**
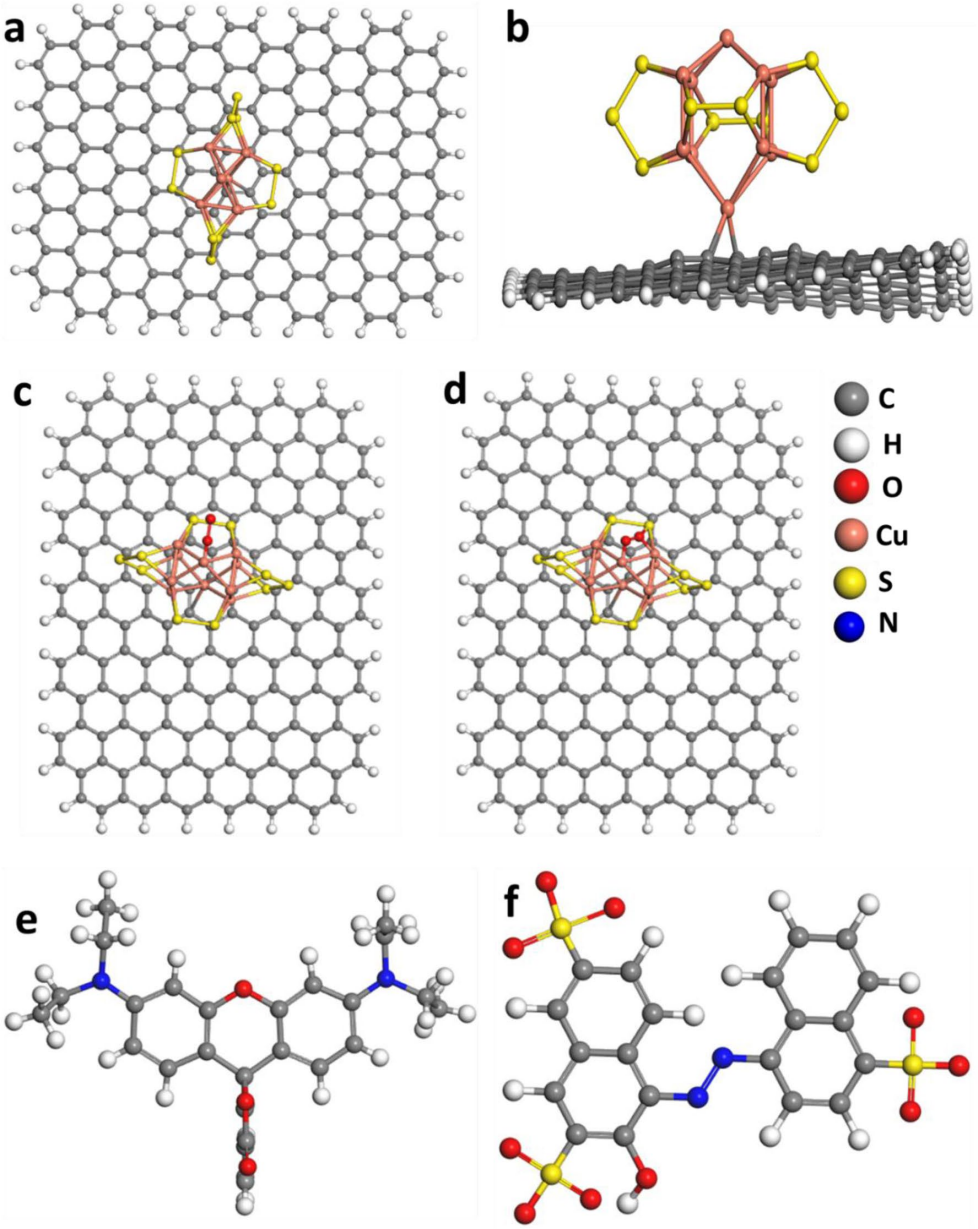
Optimized geometries for 10% CuS–rGO (**a**) top view (**b**) side view, O_2_ adsorbed (**c**) side on configuration (**d**) end on (**e**) RhB dye (**f**) amaranth dye.

**Figure 13:**
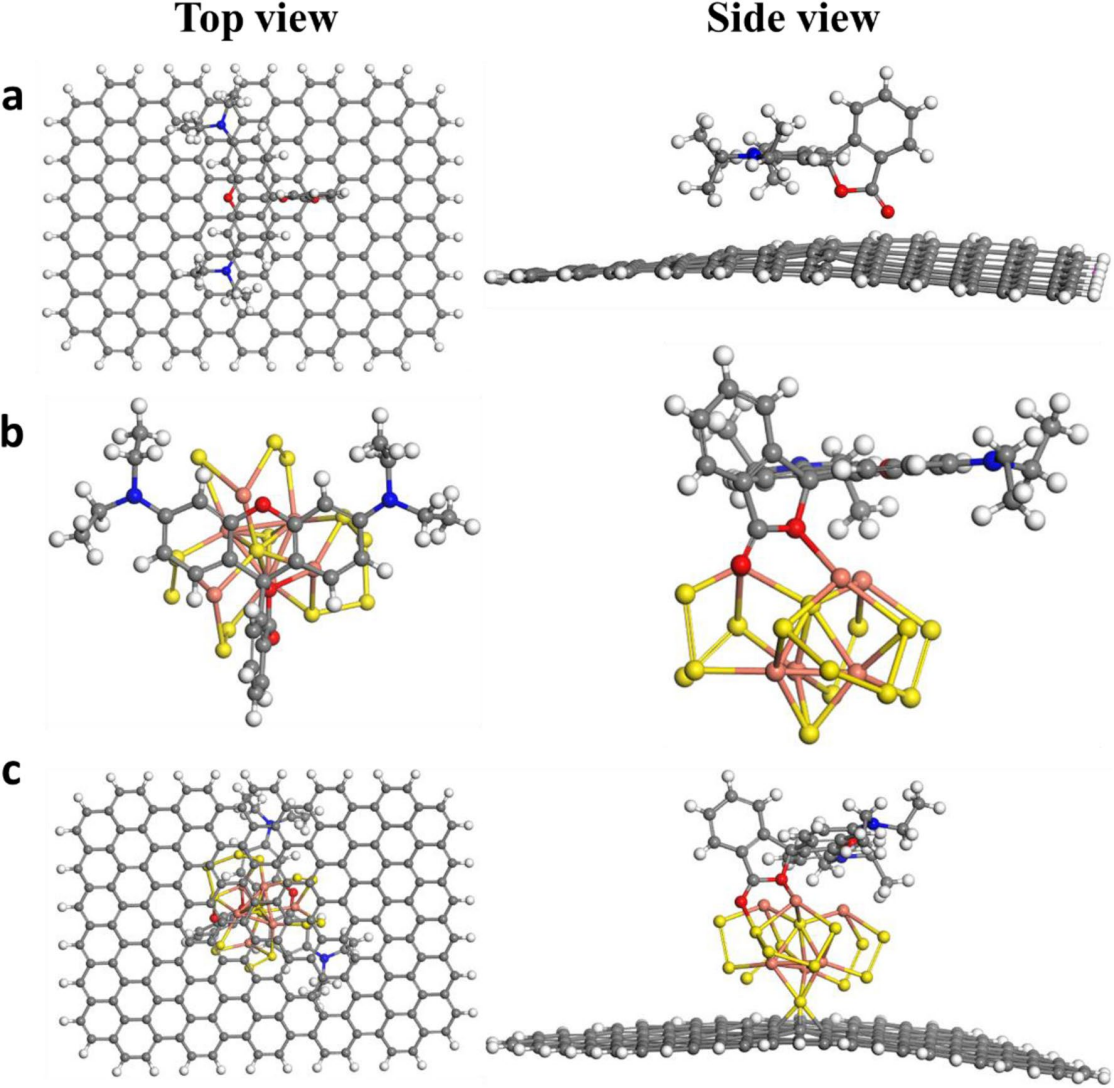
Optimized geometries of (**a**) RhB@rGO (**b**) RhB@CuS (**c**) RhB @10%CuS–rGO.

**Figure 14:**
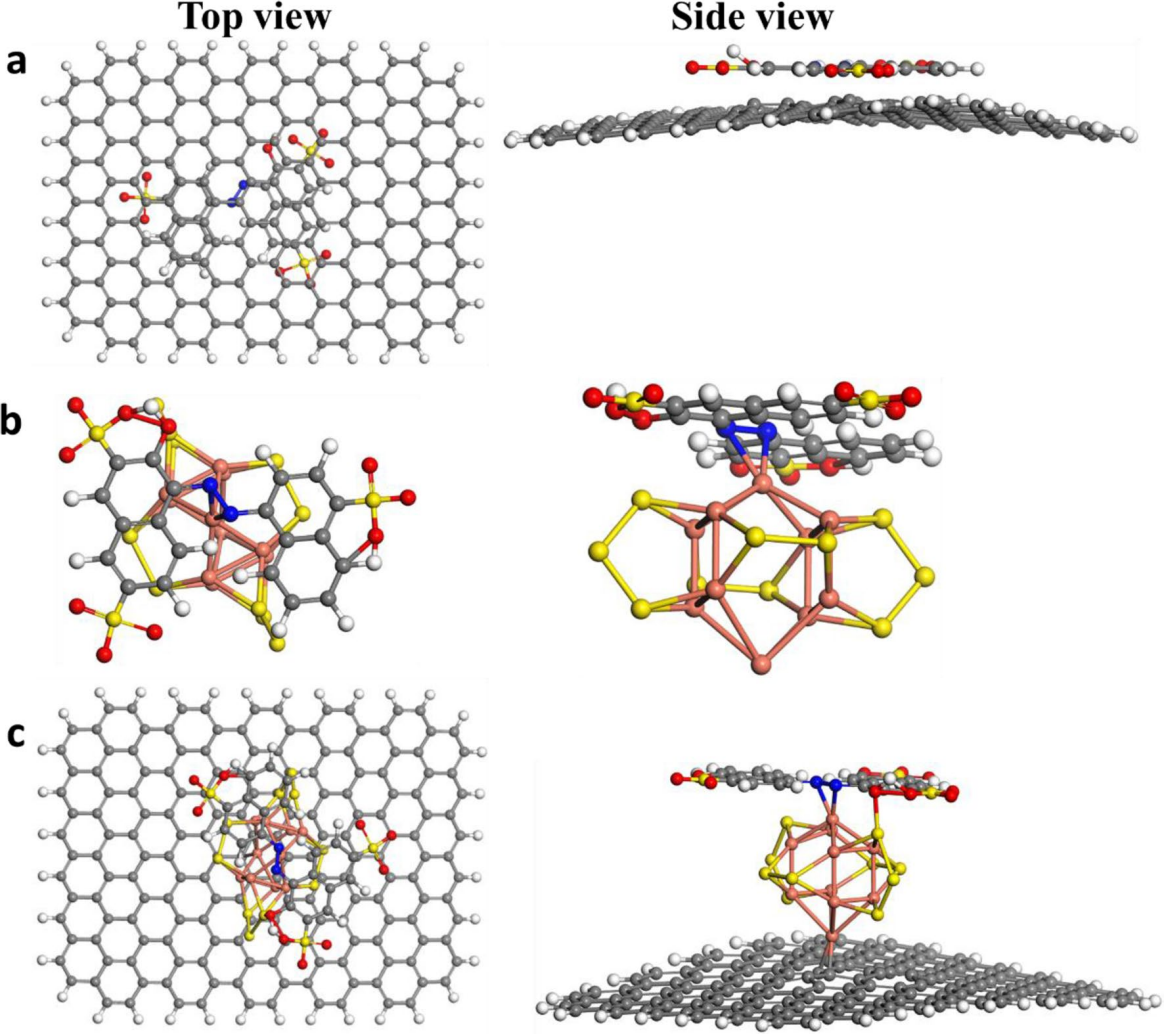
Optimized geometries of (**a**) amaranth@rGO (**b**) amaranth@CuS (**c**) amaranth@10%CuS–rGO.

To assure our experimental results DFT study was done to investigate the superoxide radicals O_2_^•−^ formation on the 10%CuS–rGO. 10%CuS–rGO was optimized as displayed in Fig. 12a, b. It is known that O_2_^•−^ has substantial function in the degradation process so the adsorption of O_2_ on 10%CuS–rGO surface was studied. The adsorption energy was calculated for two possible sites side-on and end-on at Cu atoms to be −4.01 and −2.34 eV, as shown in Fig. 12c, d respectively that means that the side-on orientation is more stable than the other configuration. In addition, elongation in the bond length between the two oxygen atoms was noticed compared to the free O_2_ as displayed in Fig. 12. They were measured to be 1.31 and 1.44 Å for the end-on and side-on configuration respectively versus 1.24 Å for O_2_ in the gas phase. From Mulliken population analysis it was observed that after adsorption on the surface O_2_ acquired −0.52 and −0.47 e^−^ for side-on and end-on arrangement respectively resulting in yielding superoxide radicals which help in the dye degradation. Also, the interactions between CuS, rGO and 10%CuS–rGO with the studied dyes (RhB and amaranth) were investigated to unravel the behavior of our catalysts towards the dye degradation. The geometries of RhB and amaranth were optimized as displayed in Fig. 12e, f respectively. After optimization of RhB dye, it was noticed from frontier molecular orbitals that it has energies values of −5.627 and −2.875 eV for HOMO (highest occupied molecular orbital and LUMO (lowest unoccupied molecular orbital), respectively. Also, it showed that the two benzene rings substituted by nitrogen atoms are electron donors and can form π–π interactions with the benzene rings of rGO, as shown in Fig. 13a. The adsorption energy of rGO with RhB was calculated to be −1.95 eV and no real chemical bond was noticed. From Mullikan charge, there was a charge transfer from rGO to RhB of about 0.05 electron. We also simulated the adsorption of RhB over CuS; the formation of chemical bonds between sulfur and copper atoms and oxygen atoms of RhB was observed, as cleared in Fig. 13b, S–O 1.92 Å and Cu–O bond length of 2.43 Å with adsorption energy of −2.87 eV. While for 10%CuS–rGO, a charge transfer from RhB to the catalyst was observed up to 0.23 electron and stronger chemical bonds were formed between sulfur and copper atoms and oxygen atoms in RhB with shorter S–O bond length of 1.75 Å and shorter Cu–O bond length of 2.01 Å, as seen in Fig. 13c. The adsorption energy of 10%CuS–rGO with RhB was measured to be −4.07 eV. The stronger adsorption energy and strong chemical bonds refer to better dye degradation^58^. Also, after optimization of amaranth on the rGO surface, the amaranth molecule was parallel on the surface without any chemical bonding with adsorption energy of −1.1 eV as shown in Fig. 14a, while amaranth with CuS, chemical bonds were formed between copper and nitrogen atoms with bond length of 2.30 Å, as cleared in Fig. 14b. For amaranth with the composite, a charge transfer from amaranth to the catalyst was estimated around 0.16 electron. The bond length between amaranth and composite reduced to 2.02 Å with a stronger adsorption energy of −3.6 eV, as shown in Fig. 14c.

## Conclusion

A simple and cheap procedure was utilized to prepare different ratios of CuS–rGO nanocomposites, and their photocatalytic efficiencies were estimated. The particle distribution obtained from TEM and SEM results clearly referred to CuS nanoparticles and rGO nanosheets. Indiscriminate distributions for particles were noticed. The CuS–rGO nanocomposites showed an effective role in the removal of both amaranth and rhodamine B dyes. DFT studies unveil the role of superoxide radicals and showed the robust binding distance and large charge transfer between the studied dyes and our composite that confirm the experimental results. The 20%CuS–rGO efficiently removes the amaranth dye after 90 min under visible light, while the 10%CuS–rGO fully degrades rhodamine B dye after 60 min under visible light. The exceptional reactivity of the nanocomposites in destructing the organic pollutants is ascribed to the positive role of rGO in transferring electrons to CuS conduction band. This electron transportation enhances the separation efficiency of the charge carrier and increases the lifetime for charge carrier production.

### Supplementary Information

Supplementary Information.

## Data Availability

The datasets used and/or analyzed during the current study available from the corresponding author on reasonable request.
