# Supplementary material for: Experimental and DFT study of photocatalytic activity of reduced graphene oxide/copper sulfide composite for removal of organic dyes from water
Source: Sci Rep. 2023 Sep 20;13:15636. doi: 10.1038/s41598-023-42680-3 (PMC10511407; doi:10.1038/s41598-023-42680-3)
Supplement: Supplementary file 1 — Supplementary Information. [file 41598_2023_42680_MOESM1_ESM.docx]

**Experimental and DFT study of photocatalytic activity of reduced graphene oxide/copper sulfide composite for removal of organic dyes from water**

Mohamed S. Sadek^*a^, Ghada E. Khedr^*b^, Michel F Abdel_messih^a^, Mohamed Abdel hay Ismail^*a^

*Corresponding authors

^a^Chemistry Department, Faculty of Science, Ain-Shams University, Cairo, Egypt

^b^Department of and Analysis and Evaluation, Egyptian Petroleum Research Institute (EPRI), Cairo 11727, Egypt


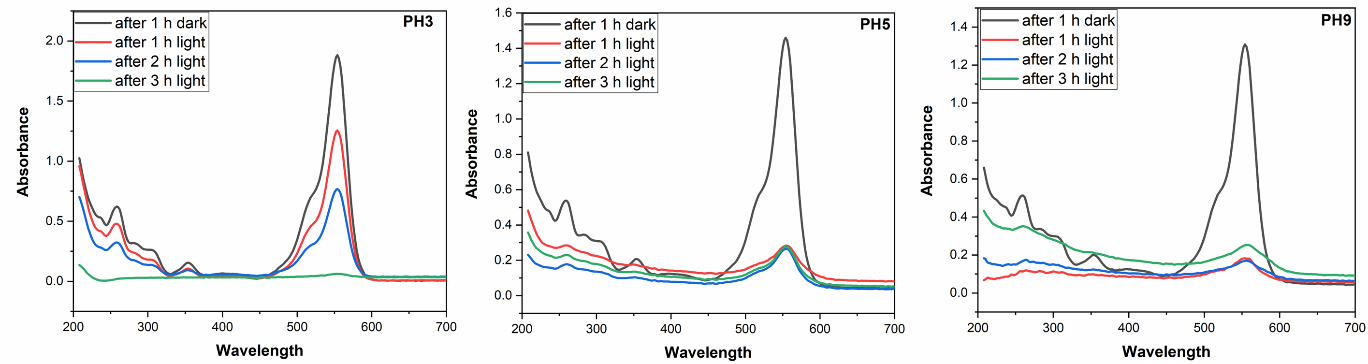


**Fig.S1**.UV-Vis absorption spectra for 10%rGO/CuS at different pH values 3, 5 and 9 in dark and in light at different times.
